# Oro-mucosal administration of oxytocin using medicated lollipops alters social attention, similar to intranasal and lingual routes: Implications for therapeutic use

**DOI:** 10.3389/fnins.2022.1022101

**Published:** 2022-10-25

**Authors:** Dan Xu, Qin Li, Qian Zhuang, Yingying Zhang, Shuxia Yao, Weihua Zhao, Keith M. Kendrick

**Affiliations:** ^1^MOE Key Laboratory for Neuroinformation, The Clinical Hospital of Chengdu Brain Science Institute, University of Electronic Science and Technology of China, Chengdu, China; ^2^School of Foreign Language, Chengdu University of Traditional Chinese Medicine, Chengdu, China; ^3^Center for Cognition and Brain Disorders, The Affiliated Hospital of Hangzhou Normal University, Hangzhou, China; ^4^Department of Molecular Psychology, Institute of Psychology and Education, Ulm University, Ulm, Germany

**Keywords:** social attention, oxytocin, top-down attention processing, anti-saccade task, oro-mucosal administration

## Abstract

A key functional effect of intranasal administration of the neuropeptide oxytocin is on top-down control of social attention. However, an oro-mucosal administration route may be better tolerated for chronic therapeutic use and evidence suggests that some functional effects of oxytocin can be mediated via peripheral routes. The current study investigated if oxytocin administered orally using medicated lollipops can both increase blood oxytocin concentrations and influence social attention and state anxiety. In a randomized, double-blind, clinical trial adult male participants received oral oxytocin (24IU) or placebo 30-min before completing a well-established anti-saccade paradigm which can assess treatment effects on both top-down and bottom-up attention. Oxytocin administration modulated top-down social attentional processing by increasing anti-saccade error rates on both social and non-social stimuli although it only increased response latencies for social cues. Anti-saccade errors were also positively associated with the proportionate increase in plasma oxytocin concentrations. A comparison analysis showed that oral oxytocin administration increased blood concentrations to a similar degree as given by lingual spray, although less than when given intranasally. Importantly, attentional and anxiolytic effects of oxytocin in the anti-saccade task were similar across intranasal, lingual, and oral administration routes. These findings demonstrate that oral administration of oxytocin, similar to via intranasal and lingual routes, can modulate top-down social attention and state anxiety and support its potential for therapeutic use. They also provide further evidence that functional effects of exogenously administered oxytocin can be mediated indirectly either by crossing the blood brain barrier or producing receptor mediated vagal stimulation, as opposed to via direct entry into the brain.

## Introduction

The hypothalamic neuropeptide oxytocin (OXT) has been demonstrated repeatedly to play an important role in social cognition and motivation in both animal models and humans (Kendrick et al., 2017). Studies on the functional effects of OXT in humans have primarily used an intranasal administration route based on evidence that it may be able to directly enter the brain via the olfactory and trigeminal nerves (see Lee et al., 2020; Quintana et al., 2021; Yao and Kendrick, 2022). While some small initial studies in young children with autism spectrum disorder have reported beneficial therapeutic effects of chronic daily intranasal OXT treatment (Yatawara et al., 2016; Parker et al., 2017) a large-scale (*n* = 277) study across a wider age-range (3–17 years) and using an escalating dose strategy of up to 80IU per day did not (Sikich et al., 2021). However, inverted U response curves for OXT and potential receptor desensitization resulting from daily dosing regimes may have contributed to variable findings (see Yao and Kendrick, 2022; Martins et al., 2022). Indeed, a more recent study has reported that using less frequent dosing with intranasal OXT (24IU every other day for 6 weeks), to reduce potential receptor desensitization effects, and given as an adjunct to positive social interactions, improved social symptoms in 44% of young autistic children using the stringent clinical reliable change index (Le et al., 2022). Another chronic dose-response study using intranasal administration of a more stable form of OXT in adults has also reported some positive effects on social symptoms in adults only when lower doses are used (Yamasue et al., 2022). Nevertheless, more large-scale clinical trials are clearly required before any therapeutic potential of oxytocin can be fully established.

There is still an ongoing debate as to the route whereby intranasal OXT produces its reported effects on brain and behavior (see Leng and Ludwig, 2016; Yao and Kendrick, 2022). Intranasal OXT administration results in increased peptide concentrations in the blood, following absorption by the capillaries in the highly vascularized nasal cavity, as well as in cerebrospinal fluid (Striepens et al., 2013). This has raised the question as to whether increased concentrations of OXT in the blood may be playing a role in observed functional effects, either by acting on its peripheral receptors and, for example, stimulating the vagal system or by binding to molecules which facilitate it crossing the blood brain barrier (BBB) (see Yao and Kendrick, 2022).

While initial studies in animal models concluded that the BBB is relatively impermeable to OXT (Mens et al., 1983; Kendrick et al., 1986), recent studies have demonstrated that it can be transported into the brain by binding to the receptor for advanced glycation end products (RAGE) in BBB endothelial cells (Yamamoto et al., 2019; Yamamoto and Higashida, 2020; Munesue et al., 2021), with *in vitro* studies also reporting the presence of this potential transport system in humans (see Higashida et al., 2022). Compared to wild-type controls, RAGE knock-out mice do not exhibit increased OXT in the brain following intranasal or subcutaneous administration (Yamamoto et al., 2019; Munesue et al., 2021). Intravenous injection of labeled OXT can also enter the cerebrospinal fluid of monkeys within 60 min (Lee et al., 2018). In humans, both intravenous and intranasal OXT mediate a decrease in regional cerebral blood flow (rCBF) in the left amygdala and anterior cingulate cortex in adult males which is associated with increased peripheral plasma OXT concentrations (Martins et al., 2020). Additionally, OXT may influence brain function by stimulating the vagus via its peripheral receptors in the heart and gastrointestinal system (Yao and Kendrick, 2022). A number of animal model studies have reported brain or behavioral effects of acute peripheral OXT administration (intravenous, intraperitoneal, or subcutaneous) (Liberzon et al., 1997; Juif and Poisbeau, 2013; Smith et al., 2019) and RAGE knockout mice in contrast to wild type controls do not show functional effects following peripheral OXT administration (Yamamoto et al., 2019) and exhibit an impaired post-partum maternal behavior (Gerasimenko et al., 2021). In humans, we have recently reported that OXT administered via lingual spray can enhance brain reward responses and arousal in response to emotional faces more effectively than following intranasal administration and effects were associated with increases in blood concentrations (Kou et al., 2021). Effects of OXT on social attention and reducing state anxiety are also equivalent following intranasal or lingual routes of administration (Zhuang et al., 2022). Additionally, early studies in autistic adults have reported that intravenous oxytocin improved social cognition and repetitive behaviors (Hollander et al., 2003, 2007). Overall, there is therefore increasing evidence that peripheral administration of OXT can produce both functional brain and behavioral effects and may be associated with the magnitude of peripheral concentration changes, although at this point it is still unresolved as to the precise mechanism(s) whereby increased peripheral concentrations of OXT influence brain and behavior.

Autistic individuals often exhibit attentional neglect of social stimuli or fail to detect salient social cues or direct attentional resources to them appropriately (Chita-Tegmark, 2016). A large number of studies in humans have demonstrated that intranasal OXT enhances attention toward social relative to non-social stimuli and salient cues such as eye-gaze in both healthy (Eckstein et al., 2019; Xu et al., 2019) and autistic individuals (Andari et al., 2016; Le et al., 2022). Our previous finding that lingually administered OXT has similar effects on modulating top-down attention to social and non-social cues as intranasal administration in an anti-saccade attentional task (Zhuang et al., 2022), suggests that an oral administration route for OXT might be used therapeutically. An intranasal administration route is potentially problematic therapeutically, especially for chronic treatment, since a number of factors can influence dosing efficiency, including application technique, size of the nasal cavity and nasal congestion. Furthermore, in young children, intranasal administration by others is not well tolerated. There is increasing interest in oro-mucosal administration strategies for pharmaceutical agents which are likely to be degraded by acidic conditions/peptidases in the gastrointestinal tract (Bastos et al., 2022). Ideally, the oro-mucosal administration should permit sufficient time for absorption of the pharmaceutical agent by the blood vessels and mucosa in the oral cavity before entering the gastrointestinal system. With OXT lingual administration, this can be achieved in adults by giving multiple sprays and instructing individuals not to swallow immediately, however for young children this is more problematic. A potential more suitable alternative for children is to use a medicated lollipop approach where the pharmacological agent, OXT in this case, is freeze-dried onto the surface of the lollipop and can slowly dissolve into the mouth during licking. We therefore took the novel step of using this approach to administer OXT in adults as a proof of principle study to determine both its efficacy for increasing blood OXT concentrations and for modulating top-down social and non-social attention.

To allow comparison with previous intranasal (Xu et al., 2019) and lingual spray (Zhuang et al., 2022) OXT administration studies, the current oral OXT administration study also used an adapted version of the emotional anti-saccade paradigm to examine its effects in modulating top-down and bottom-up social and non-social attentional processing. In this paradigm, effects on volitional top-down attention are evidenced by errors and saccade latencies when subjects are required to look away from stimuli (anti-saccades) or on automatic bottom up attention when subjects are required to look toward stimuli (pro-saccades). The weaker effects of OXT on non-social top-down attention observed in previous studies (Xu et al., 2019; Zhuang et al., 2021, 2022) might have been due to greater complexity of faces compared to simple oval shapes. We therefore adapted the paradigm to include images of houses inside the oval shapes which are classically used as controls for face images (Arkush et al., 2013; Oruc et al., 2018). The current study also measured blood concentrations of OXT before and after oral administration both in the main study and at more frequent time intervals in a separate pilot study on a different cohort of subjects. As in previous studies (Xu et al., 2019; Zhuang et al., 2022), we also measured effects of OXT on reducing state anxiety. Overall, we hypothesized that oral OXT administered via medicated lollipops would produce similar effects on top-down social attention as with intranasal (Xu et al., 2019) and lingual (Zhuang et al., 2022) routes and that these would be associated with blood OXT concentrations. We also hypothesized that we would observe greater effects on top-down attention involving the more complex non-social stimuli. Finally, we hypothesized that orally administered OXT would produce a similar profile of increased concentrations in blood as lingual administration (Kou et al., 2021) and would reduce state anxiety similar to both intranasal (Xu et al., 2019) and lingual (Zhuang et al., 2022) OXT.

## Materials and methods

### Participants, validation of oral administration approach and questionnaires

**FIGURE 1:**
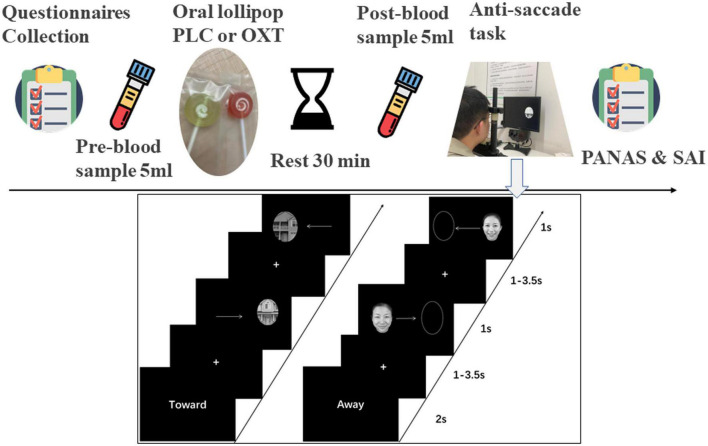
Experimental procedure for questionnaires, oral oxytocin administration and blood sampling **(top)** and the emotional anti-saccade paradigm **(below)**. “Toward” indicates the pro-saccade task where participants have to make a saccade toward the stimulus and “Away” indicates the anti-saccade task where participants have to make a saccade away from the side where the stimulus appears.

Seventy-two healthy male college students aged 18–30 years (Mean ± SEM = 21.15 ± 0.23) were recruited in a randomized, double-blind, placebo-controlled study (see Figure 1). An *a priori* power analysis showed that this would achieve 80% power for α = 0.05 and a medium effect size (*f* = 0.25) (G*Power). Participants randomly received either oro-mucosal treatment with OXT using commercial sugar-free lollipops (24IU, *n* = 35, age: Mean ± SEM = 21.42 ± 0.37) or placebo (PLC) treated sugar-free lollipops (*n* = 35, age: Mean ± SEM = 20.9 ± 0.27). One surface of the lollipop was coated with OXT (24IU OXT dissolved in 0.1 ml sterile water, 0.9% sodium chloride and glycerol and then freeze-dried) or PLC (0.1 ml of same solution but without OXT and then freeze-dried). Random allocation to treatment groups was computer generated. See Supplementary Figure 1 for consolidated standards of reporting trials (CONSORT) flow chart. All subjects were required not to consume caffeine or alcohol for 24 h prior to the experiment. Only male participants were included to match with previous oral and intranasal OXT studies using the same task and given the objective of establishing whether oral OXT might be effective therapeutically in autism, which is primarily a disorder affecting males (Xu et al., 2019; Kou et al., 2021). Exclusion criteria for all participants were as follows: (i) Has had or is suffering from a neurological or psychiatric disorder; (ii) Use of any psychotropic drugs, including nicotine. All participants volunteering to take part were informed about details of the experiment before signing an informed consent form. All experimental procedures in this study followed the latest version of the Declaration of Helsinki and were also approved by the ethics committee of the University of Electronic Science and Technology, and pre-registered on the clinical trial website (NCT: https://www.clinicaltrials.gov, ID: NCT05444738).

In order to validate our oral OXT administration protocol using a medicated lollipop approach a pilot study on 15 adult male subjects (oral OXT lollipop: *n* = 10, oral PLC lollipop: *n* = 5) was performed. This investigated the pharmacodynamics of the 24IU medicated lollipop on plasma and saliva OXT concentrations over a period of 2 h. Blood samples were taken every 15 min via an indwelling catheter, two baselines before oral administration and eight samples following it; saliva samples were taken by a passive drool method before administration and 30 and 120 min after. In order to ensure that OXT freeze-dried on the surface of the lollipop dissolved in the mouth and absorbed by capillaries we asked subjects to lick, but not bite, the lollipop for 3 min.

To control for potential confounding effects of between-group differences in mood, personality and past experiences before treatment, all subjects in the main experiment first completed a set of validated questionnaires in Chinese. These included measures of depression [Beck Depression Inventory-II (BDI)–Beck et al., 1996], anxiety [State-Trait Anxiety Inventory (STAI)–Spielberger et al., 1971; Liebowitz Social Anxiety Scale (LSAS)–Mattick and Clarke, 1998], autistic traits [Autism Spectrum Quotient (ASQ)– Baron-Cohen et al., 2001 and Social Responsiveness Scale-2 (SRS-2)–Constantino and Gruber, 2012], early life experience [Childhood Trauma Questionnaire (CTQ)–Bernstein et al., 1998], alexithymia [Toronto Alexithymia Scale (TAS-20)–Bagby et al., 1994], emotion regulation [Emotional Regulation Questionnaire (ERQ)–Garnefski and Kraaij, 2007], behavioral response control [Behavioral Activation and Inhibition Scale (BAS/BIS)–Carver and White, 1994], and mood [Positive and Negative Affect Schedule (PANAS-18)–Watson et al., 1988]. Subjects then had a 5 ml blood sample taken by venipuncture for measurement of baseline OXT and received either OXT or PLC via a lollipop. A second blood sample was taken 30 min after administration to measure increased plasma OXT concentrations. After a short break, subjects were seated comfortably in front of an eye-tracking machine (Eye-link 1000) and after a brief practice of the anti-saccade task started the main task 40–45 min after oral treatment. Immediately after the task, subjects completed the PANAS and STAI questionnaires again to assess potential treatment and task effects of mood and state anxiety. Subjects were finally asked to guess which treatment they had received and analysis revealed that they were unable to do better than chance (χ^2^ = 0.19, *p* = 0.66).

### Anti-saccade paradigm

An adapted version of the anti-saccade paradigm from our previous studies was used (Xu et al., 2019; Zhuang et al., 2021, 2022), with a total of 576 trials in 14 blocks. The first two blocks presented non-social stimuli (one anti-saccade and one pro-saccade block with 48 trials per block). However, in contrast to our previous studies where only a simple empty oval shapes were used here we used oval shapes incorporating pictures of houses taken from different angles and varying shades of gray in order to better match the complexity of the social facial stimuli **(see** Figure 1). The next 12 blocks presented social stimuli (emotional faces of angry, fear, happy, sad, and neutral of four males and four females) including six pro-saccade and six anti-saccade blocks with eight stimuli per block. Each block started with the presentation of cue words (“Toward”–i.e., pro-saccade or “Away”–i.e., anti-saccade) for 2,000 ms, followed by a “+” fixation on the screen (duration: 1,000–3,500 ms jittered). Next, a stimulus appeared on either the left or right side of the screen randomly for 1,000 ms. If the cue word was “Toward,” the subject had to make a saccade toward the stimulus and if “Away” they needed to make a saccade away from it as quickly as possible. Subjects were required to complete the task quickly while maintaining accuracy. A 30 s break was allowed at the end of each block. Subjects initially received 32 practice trials (16 social and 16 non-social) on the task to reduce avoid potential problems with higher error-rates in initial trials (see Smyrnis et al., 2002).

### Eye movement recording and processing

Eye movement data recording was carried out using an EyeLink 1000 Plus system (SR Research, Ottawa, ON, Canada) in monocular mode with a 2,000 Hz sampling rate and a 1,024 × 768 screen resolution. The fixed standard distance from the subject’s eyes to the screen was set to 57 cm (using a fixed chin rest), and a nine-point calibration was conducted prior to start each block to obtain high quality eye-tracking data. The EyeLink Data Viewer 3.1 was used to export and pre-process the raw eye movement data. In line with previous studies, trials with latencies <70 or >700 ms and saccade velocity lower than 30°/s were discarded (García-Blanco et al., 2013; Xu et al., 2019; Zhuang et al., 2021, 2022). Seven subjects could not complete the experiment due to technical problems (OXT: 4; PLC: 3) and were therefore excluded. An error was defined as subjects making their first saccade in the opposite direction to the one they had been instructed to do. Finally, the mean error rate and latencies of correct saccade trials during both anti- and pro-saccade conditions were calculated and served as primary behavioral indices.

### Blood sampling and oxytocin assays

Blood sampling and plasma OXT measurement. Five-milliliter venous blood samples were collected into EDTA tubes (two tubes for pre-treatment, two tubes for post-treatment) and immediately cooled and centrifuged at 1,600 × *g* for 15 min at 4°C. Sampling, handling, and OXT assay protocols are as in previous studies (see Kou et al., 2021; Le et al., 2022). Samples were run in duplicate and were subjected to a prior extraction step followed by an ELISA (ENZO Life Sciences, NY, USA). There has been some controversy concerning the validity of ELISA results for plasma OT (Leng and Ludwig, 2016) but we incorporated both the recommended extraction step and recovery of spiked samples to address this issue and our mean basal concentrations are in the expected normal range (i.e., <10 pg/ml).

### Statistical analyses

Statistical analyses were either performed using SPSS 25.0 (SPSS Inc., Chicago, IL, USA) or, where Shapiro–Wilk tests indicated a non-normal distribution, using nparLD software (Noguchi et al., 2012) for R-based non-parametric tests. To determine the effects of OXT, 2 (social/non-social stimuli) × 2 (pro-/anti-saccade) × 2 (OXT/PLC) ANOVAs were used for both error rates and response latencies. To further explore the effect of OXT on individual emotional expressions, 6 (anger/fear/happy/neutral/sad/shape) × 2 (pro-/anti-saccade) × 2 (OXT/PLC) ANOVAs were conducted on both error rate and response latencies. In addition, we performed correlation analyses between plasma OXT concentration changes (%) and behavior using Pearson to establish whether functional effects of oral OXT on modulating social attention and state anxiety (SAI) were associated with them. Correlations between SAI scores and performance on the anti-saccade task were also analyzed.

In a secondary analysis, a comparison across the effects of OXT administered intranasally (Xu et al., 2019), lingually (Kou et al., 2021), and orally (medicated lollipop) on both anti-saccade error rates and response latencies was performed using ANOVAs. In line with recent recommendations in the field (Quintana, 2022), the robustness of non-significant findings was additionally assessed using Bayesian analysis (JASP, version 0.14.1.0).^1^ In, addition, we also conducted an exploratory analysis of changes in plasma OXT concentrations following administration of 24IU OXT by intranasal, lingual or oral routes using a non-parametric ANOVA due to a non-normal distribution of the data. For parametric ANOVAs, appropriate Bonferroni-corrected comparisons were employed within SPSS to control for multiple comparisons or for R-based non-parametric analyses Bonferroni-corrected Mann–Whitney *U*-tests were conducted for multiple comparisons.

## Results

### Effects of oral oxytocin administration *via* medicated lollipop on plasma and saliva oxytocin concentrations (pilot experiment)

Effects of oral administration of 24IU OXT by medicated lollipop (*n* = 10) on proportionate increases in plasma and saliva OXT concentrations were compared to those following PLC (*n* = 5). For plasma samples, data were not normally distributed and so a non-parametric ANOVA-type analysis was performed with treatment (OXT/PLC) and time-point (0/15/30/45/60/75/90/105/120 min) as factors. There were significant main effects of both treatment (*F*_1_, _6_._90_ = 5.59, *p* = 0.05) and time-point (*F*_3_._10_, _8_ = 2.82, *p* = 0.036) although not the treatment × time-point interaction (*F*_2_._32_, _8_ = 1.22, *p* = 0.306). Exploratory *post-hoc* tests using Mann–Whitney showed that the percentage increase in OXT was significant in the OXT vs. PLC group at both 15 min (*z* = 2.511, *p* = 0.0121) and 30 min (*z* = 2.7557, *p* = 0.006) post-administration time points. Wilcoxon tests were also used to compare between baseline (time-point: 0) and other time-points within the OXT group and *p*-values Bonferroni-corrected. OXT concentrations in the OXT group were significantly increased at both 15 (*p* = 0.001) and 30 (*p* = 0.001) min time-points (**see** Supplementary Figure 2). A similar analysis of OXT concentrations in saliva (time-points, −15, +30, and +120 min) revealed large significant increases at both +30 min (>30-fold) and +120 min (5-fold) (both *z* = −2.886, *p* = 0.004, Wilcoxon) compared to baseline **(see** Supplementary Figure 2).

### Potential confounders

Independent *t*-tests showed no significant differences between OXT and PLC groups across questionnaire scores before treatment (all *ps* > 0.05–see Table 1) indicating that the two groups were well-matched.

**TABLE 1:** Mean ± SD questionnaire scores in OXT and PLC groups before and after attention task.

Questionnaires	OXT	PLC	*t*-value	*P*-value
**Before task**				
PANAS_P	22.34 (6.88)	20.88 (6.32)	0.895	0.374
PANAS_N	22.66 (5.37)	21.24 (5.79)	1.020	0.311
STAI (state)	39.87 (7.75)	40.09 (8.80)	–0.106	0.916
STAI (trait)	42.10 (7.05)	43.47 (8.58)	–0.692	0.492
BDI-II	7.50 (7.43)	10.15 (6.87)	–1.496	0.140
LSAS (avoid)	20.06 (11.23)	22.79 (11.73)	–0.957	0.342
LSAS (fear)	24.41 (12.36)	26.42 (12.92)	–0.643	0.522
BAS-reward responsiveness	6.85 (1.95)	7.15 (1.75)	–0.669	0.506
BAS-drive	7.75 (2.14)	8.06 (1.98)	–0.607	0.546
BAS-fun seeking	9.81 (2.19)	9.88 (2.41)	–0.116	0.908
BIS-behavioral inhibition	15.22 (2.32)	15.49 (2.70)	–0.425	0.672
ERQ	46.00 (6.06)	45.76 (7.75)	0.140	0.889
ASQ	21 (4.09)	21.273 (5.61)	–0.223	0.824
TAS	49.78 (8.34)	53.39 (9.23)	–1.654	0.103
CTQ	41.53 (8.42)	40.67 (6.13)	0.475	0.637
**Post-task**				
PANAS_P	16.68 (3.75)##	16.67 (3.40)##	0.012	0.990
PANAS_N	16.23 (3.10)##	16.06 (2.76)##	0.226	0.822
STAI (state)	34.07 (6.79)##	38.13 (8.50)	–2.068	0.043*

PANAS, positive and negative affect schedule; STAI, State-Trait anxiety inventory; BDI, Beck Depression Inventory; LSAS, Liebowitz Social Anxiety Scale; BAS/BIS, behavioral inhibition/activation system scale; ERQ, Emotion Regulation Questionnaire; ASQ, Autism Spectrum Quotient; TAS, Toronto Alexithymia Scale; CTQ, Childhood Trauma Questionnaire. *p < 0.05 OXT vs. PLC group (*t*-test) and ##*p* < 0.01 vs. pre-task (paired *t*-test).

### Oral oxytocin effects on saccade error rate and response latency

**FIGURE 2:**
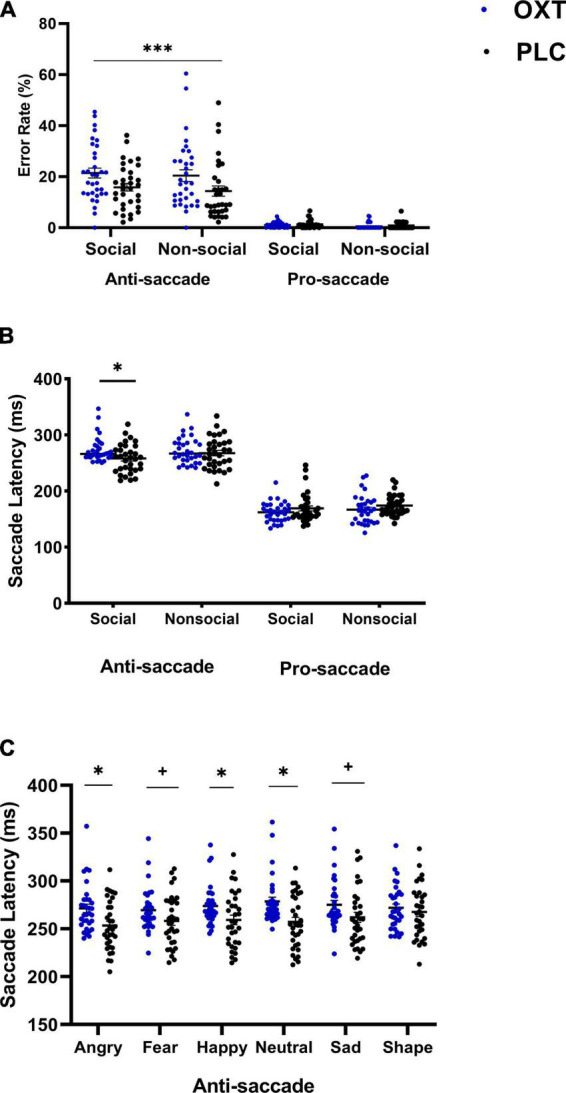
The effects of oral (lollipop) oxytocin (OXT) on error rates and saccade latencies in the anti-saccade task. Oral OXT increased **(A)** anti-saccade and pro-saccade error rates compared for OXT and PLC groups and for social and non-social stimuli (***p < 0.001 treatment × task interaction, indicating that OXT increased anti- but not pro-saccade errors for both social and non-social stimuli), and **(B)** for anti-saccade and pro-saccade response latencies for social and non-social stimuli **(C)** increased anti-saccade response latencies were seen across face emotions, and differed significantly from non-social stimuli. For panels **(B,C)**,*p < 0.05 and two-tailed and +*p* < 0.05 one-tailed *post hoc* Bonferroni-corrected treatment effect for OXT vs. PLC.

For error rates, data were not normally distributed and so non-parametric statistics were used for analysis. The treatment (OXT/PLC) × condition (social/non-social) × task (pro-/anti-saccade) mixed ANOVA-type on error rate showed a significant treatment × task interaction (*F*_1_, _8_ = 11.47, *p* = 0.007). *Post hoc* comparisons (Mann–Whitney *U*-test) indicating that OXT only increased error rates in the anti-saccade but not pro-saccade task (anti-saccade: PLC: Mean ± SEM = 15.1% ± 1.70, OXT: Mean ± SEM = 21.6% ± 1.7, *z* = 3.45, *p* = 0.001; pro-saccade: PLC: Mean ± SEM = 1.10% ± 0.20, OXT: Mean ± SEM = 0.85% ± 0.18, *z* = 0.99, *p* = 0.3222) (see Figure 2A). There was also a significant main effect of task (*F*_1_, _8_ = 770.68, *p* < 0.001), with higher error rates for anti-saccades compared to pro-saccades, and a condition × task interaction (*F*_1_, _8_ = 4.13, *p* = 0.042). *Post-hoc* tests showed that errors were higher for pro-saccades in the non-social condition (*z* = 3.585, *p* = 0.001). No other significant main or interaction effects of condition were found (all *ps* > 0.131).

In a mixed non-parametric ANOVA including stimuli as a factor, no significant three-way interaction was found for treatment (OXT/PLC) × stimuli (angry/sad/fearful/happy/neutral/shape) × task (pro-/anti-saccade) on error rates (*F*_4_._39_, _8_ = 1.15, *p* = 0.332), indicating no differential treatment effects across the individual social and non-social stimuli. Task and stimuli main effects and a treatment × task interaction were all significant (all *ps* < 0.024), with the *post hoc* comparisons again showing a greater number of errors in the anti-saccade task following OXT (*z* = −5.24, *p* < 0.001).

For response latency, the condition (social/non-social) × treatment (OXT/PLC) × task (pro-/anti-saccade) mixed parametric ANOVA revealed a significant three-way interaction (*F*_1_, _63_ = 4.19, *p* = 0.045, η*_*p*_*^2^ = 0.062). *Post hoc* Bonferroni-corrected comparisons indicated that response latencies in the anti-saccade task for social stimuli were significantly longer in the OXT than in the PLC group (*F*_1_, _63_ = 6.84, *p* = 0.011, η*_*p*_*^2^ = 0.098, see Figure 2B). There was also a two-way treatment × task interaction (*F*_1_, _63_ = 9.33, *p* = 0.003, η*_*p*_*^2^ = 0.129). However, *post hoc* analysis showed the interaction effect was primarily driven by task rather than treatment with both treatment groups showing faster response latencies for pro-saccades relative to anti-saccades (OXT: anti-saccade: Mean ± SEM = 272.81 ± 4.18, pro-saccade: Mean ± SEM = 164.52 ± 3.53, *p* < 0.001, Cohen’s *d* = 4.89; PLC: anti-saccade: Mean ± SEM = 262.82 ± 4.11, pro-saccade: Mean ± SEM = 171.608 ± 3.47, *p* < 0.001; Cohen’s *d* = 3.68).

The mixed ANOVA including stimuli as a factor revealed a significant three-way (stimulus × treatment × task) interaction (*F*_5_, _315_ = 2.63, *p* = 0.042, η*_*p*_*^2^ = 0.04). The *post hoc* analysis indicated that anti-saccade response latencies in the OXT group were longer for angry (*p* = 0.006), fear (*p* = 0.073), sad (*p* = 0.063), happy (*p* = 0.025), and neutral (*p* = 0.002) expression faces but not for non-social shapes (*p* = 0.498). There were no differences between individual stimuli (see Figure 2C). In addition, there was a significant task × treatment interaction (*F*_1_, _63_ = 11.22, *p* = 0.001, η*_*p*_*^2^ = 0.15) with *post-hoc* tests indicating that anti-saccade latencies were longer in the OXT compared to the PLC group (OXT: Mean ± SEM = 273.77 ± 4.18, PLC: Mean ± SEM = 259.62 ± 4.12, *p* = 0.02, Cohen’s *d* = 0.55). The main effects of stimuli and task were also significant (*F*_5_, _315_ = 5.26, *p* = 0.001, η*_*p*_*^2^ = 0.08; *F*_1_, _63_ = 1057.74, *p* < 0.001, η*_*p*_*^2^ = 0.94) with pairwise comparisons of stimuli showing saccade latencies for angry (*p* = 0.030) and fear (*p* = 0.020) faces were faster than those for shapes.

### Oral oxytocin effects on anxiety and mood

After treatment and performing the task there was a significant reduction in state anxiety (SAI) scores in the OXT compared to the PLC group (*t*-test, *p* = 0.043) (see Table 1). Paired *t*-tests conducted on pre- compared to post-task SAI scores showed that SAI scores were significantly decreased in the oral OXT group (*t* = −3.30, *p* = 0.0026) but not the PLC group (*t* = −1.251, *p* = 0.22). For mood scores both PANAS positive and negative scores were significantly reduced in both the OXT (positive *t* = −5.14, *p* < 0.001; negative *t* = −2.49, *p* < 0.001) and PLC (positive *t* = −3.62, *p* = 0.001; negative *t* = −4.55, *p* < 0.001) groups but there were no differences between treatments (see Table 1).

### Plasma oxytocin concentration changes in the main experiment and association with attention and state anxiety

**FIGURE 3:**
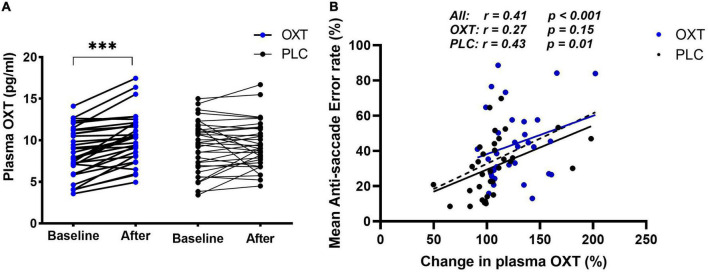
Effect of oral oxytocin (OXT) on plasma concentration of OXT in the main experiment **(A,B)** correlations between % anti-saccade errors and % change in plasma OXT 30 min after oral OXT or PLC administration. The correlation data points and regression lines for OXT (blue) and PLC (black) groups are shown both separately and combined (dashed line). ***p < 0.001 *t*-test.

A 2 × 2 mixed parametric ANOVA was performed with plasma OXT concentration as the dependent variable, and time point (time 0 and +30 min) and treatment group (OXT/PLC) as independent variables. Results showed a significant main effect of time point (*F*_1_, _63_ = 21.76, *p* < 0.001, η*_*p*_*^2^ = 0.26) but not treatment (*F*_1_, _63_ = 0.007, *p* = 0.93) and a treatment × time point interaction (*F*_1_, _63_ = 14.75, *p* = 0.001, η*_*p*_*^2^ = 0.19, see Figure 3A). The Bonferroni-corrected *post-hoc* tests revealed a significantly greater increase in plasma OXT concentration in the OXT compared to the PLC group (*p* < 0.001). A further mixed ANOVA with SAI score as the dependent variable and time (before/post-task) and treatment (OXT/PLC) as factors revealed a significant main effect of time (*F*_1_, _62_ = 9.74, *p* = 0.003, η*_*p*_*^2^ = 1.36) with individuals in both treatment groups reporting reduced SAI scores after the test. A similar ANOVA analysis on PANAS positive (*F*_1_, _62_ = 26.56, *p* < 0.001, η*_*p*_*^2^ = 0.3) and negative (*F*_1_, _62_ = 52.25, *p* < 0.001, η*_*p*_*^2^ = 0.46) scores also revealed main effects of time due to a reduction in scores post-task (see Table 1). There were no significant time × treatment interactions for SAI or PANAS scores (all *ps* > 0.142).

Pearson correlation analysis indicated that both OXT (*r* = 0.27, *p* = 0.15) and PLC (*r* = 0.43, *p* = 0.02) groups had a positive correlation between the percentage changes in OXT concentrations 30-min after treatment and mean anti-saccade errors, although this only reached significance in the PLC group. There was no difference between the two groups however (Fisher’s *z* = −0.651, *p* = 0.258) and so to increase statistical power, we therefore combined treatment groups and observed a robust positive correlation between percentage changes in OXT concentrations and anti-saccade errors (*r* = 0.411, *p* < 0.001) (see Figure 3B) but not latencies (*r* = 0.077, *p* = 0.542) or post-task reductions in state anxiety (*r* = −0.214, *p* = 0.095). There were no significant associations with post-task reductions in PANAS scores (*ps* > 0.553) or between task performance and pre- or post-task SAI scores (all *rs* < −0.21, *ps* > 0.25).

### Comparison of different routes of oxytocin administration on attention control and state anxiety

**FIGURE 4:**
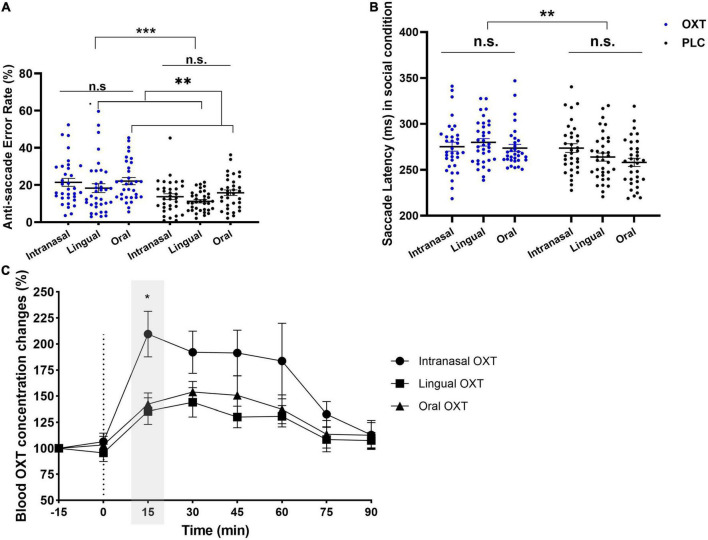
The comparative results of intranasal, lingual, and oral on error rates, saccade latencies, and peripheral OXT concentrations. **(A)** Significant treatment (*H* = 9.40, *p* = 0.009) and route (*H* = 16.16, *p* < 0.001) main effects to social cues on anti-saccade errors were observed, and oral administration produced higher errors than lingual (*p*_adj_. = 0.008). **(B)** Bonferroni-corrected pairwise comparisons of condition × treatment (*F*_1_, _195_ = 18.98, *p* < 0.001, η*_*p*_*^2^ = 0.089) showed longer anti-saccade latencies to social cues in OXT group compared to PLC (*p* = 0.002). **(C)** Changes in peripheral OXT concentration only showed a time main effect (*F*_4_._95_, _8_ = 52.43, *p* < 0.001), and the exploratory Bonferroni-corrected multiple comparison showed OXT concentration in intranasal was significantly higher than lingual (*z* = 2.413; *p*_Bonferroni–corrected_ < 0.05) at 15 min. *p < 0.05, **p < 0.01, ***p < 0.001.

A total of 201 subjects from the three studies were included in this analysis resulting in an estimate of 78% power achieved by the 3-factor ANOVAs (G*Power) for a medium effect size (*f* = 0.25) and α = 0.05. Given that significant effects of all three routes of OXT administration (intranasal, lingual, and oral) were on top-down attention control (i.e., anti-saccade errors and response latencies) we only compared the relative efficacy of the different routes in the anti-saccade task. A non-parametric route (intranasal/lingual/oral) × treatment (OXT/PLC) × condition (social/non-social) mixed ANOVA-type test on anti-saccade error rate showed significant main effects of condition (*F*_1_, _8_ = 61.50, *p* < 0.001), treatment (*F*_1_, _185_._79_ = 22.48, *p* < 0.001) and route (*F*_2_, _185_._79_ = 10.60, *p* < 0.001) and a route × condition interaction (*F*_1_._99_, _8_ = 5.45, *p* = 0.004). *Post hoc* Bonferroni-corrected Mann–Whitney *U*-tests showed anti-saccade error rates were higher for oral compared to lingual administration for social cues (*z* = −3.045; *p* < 0.05) and compared to lingual (*z* = −4.287; *p* < 0.001) and intranasal (*z* = −3.922; *p* < 0.001) administration for non-social cues. No other significant main or interaction effects were observed (all *ps* > 0.595). Given that OXT administration increased both social and non-social anti-saccade error rates we additionally compared its efficacy across routes specifically for social anti-saccade error rates on social stimuli. A Scheirer–Ray–Hare test for route (intranasal/lingual/oral) × treatment (OXT/PLC) was performed using the R-package “rcompanion,” and results showed significant main effects of treatment (*H* = 9.40, *p* = 0.009) and route (*H* = 16.16, *p* < 0.001). Bonferroni-corrected Dunn tests showed oral administration produced higher anti-saccade error rates compared to lingual (*p*_*adj.*_ = 0.008), but the interaction between them was not significant (*H* = 0.14, *p* = 0.93) (see Figure 4A).

To further investigate potential administration route-dependent effects of OXT effect on specific stimuli a mixed ANOVA with stimuli (angry/fear/happy/neutral/sad/shapes) and route (intranasal/lingual/oral lollipop) and treatment (OXT/PLC) as factors was performed for anti-saccade error rates. There were main effects of stimuli (*F*_4_._07_, _8_ = 20.51, *p* < 0.001), treatment (*F*_1_, _184_._19_ = 22.25, *p* < 0.001) and route (*F*_2_, _184_._19_ = 6.29, *p* = 0.002), and a stimuli × route interaction (*F*_7_._97_, _8_ = 2.89, *p* = 0.003). The *Post hoc* Bonferroni-corrected tests indicated that error rates for shape stimuli following oral administration were significantly higher than for lingual (*z* = −4.287, *p* < 0.001) and intranasal (*z* = −3.922, *p* < 0.01). There were no other significant interactions (all *ps* > 0.638). Overall, these results suggest that OXT evoked similar increases in anti-saccade errors across different facial expressions following all three routes of administration. However, the inclusion of more complex non-social stimuli in the current oral administration study increased the number of anti-saccade errors compared to the previous lingual and intranasal studies using simpler shape stimuli.

A similar route × treatment × condition mixed ANOVA analysis for anti-saccade response latencies only revealed a condition × treatment interaction (*F*_1_, _195_ = 18.98, *p* < 0.001, η*_*p*_*^2^ = 0.089). *Post hoc* Bonferroni-corrected tests indicated that response latencies were significant longer in the OXT than PLC group for the social stimuli condition (see Figure 4B). There were no other significant main or interaction effects (all *ps* > 0.126). A stimuli × treatment × route ANOVA again revealed a main effect of treatment (*F*_1_, _195_ = 6.250, *p* = 0.013, η*_*p*_*^2^ = 0.031) and stimuli × treatment interaction (*F*_5_, _975_ = 12.48, *p* < 0.001, η*_*p*_*^2^ = 0.060). *Post hoc* Bonferroni-corrected tests showed response latencies in the OXT group were longer than in the PLC group across all stimuli (all *ps* < 0.032). No other significant main effects or interactions were observed (all *ps* > 0.116).

A route × treatment ANOVA performed on pre- vs. post-task differences in SAI scores also revealed a main effect of treatment (*F*_2_, _192_ = 3.296, *p* = 0.035 one-tailed, η*_*p*_*^2^ = 0.016) and no significant route × treatment interaction (*F*_2_, _192_ = 0.497, *p* = 0.609) indicating an absence of differences between the routes of OXT administration.

In accordance with recent recommendations in the field (Quintana, 2022) we additionally examined the robustness of the non-significant findings for the comparison between different routes of OXT administration by performing Bayesian ANOVAs on anti-saccade errors and latencies for social stimuli. Results showed a satisfactory moderate fit for anti-saccade errors (BF_01_ = 5.29) and latencies (BF_01_ = 6.84). Similarly, for SAI scores the Bayes analysis revealed a moderate fit (BF_01_ = 8.10). Thus, overall this Bayesian analysis confirmed the lack of any administration route-dependent effects of OXT on top-down attention or state anxiety.

### Exploratory comparison of changes in peripheral oxytocin concentrations following intranasal, lingual or oral administration

A comparison of changes plasma in OXT concentrations over time with different routes of administration was also carried out, with data for intranasal (*n* = 15) and lingual (*n* = 10) routes from a previous report (Kou et al., 2021). The route × time non-parametric ANOVA showed a significant main effect of time (*F*_4_._95_, _8_ = 52.43, *p* < 0.001). No other effects were significant (all *ps* > 0.211). The Bonferroni-corrected multiple comparison showed OXT concentrations following intranasal administration were significantly higher than after lingual (*z* = 2.413; *p* < 0.05) administration (see Figure 4C).

## Discussion

In an initial pilot experiment, we confirmed that administration of OXT orally using a medicated lollipop approach significantly increased plasma OXT concentrations at 15 and 30 min after administration and concentrations in saliva were greatly increased after both 30 min and 2 h. The profile of increased plasma OXT concentrations was similar to that previously reported following lingual administration, although lower than initially (15 min) after intranasal administration (Kou et al., 2021). In the main experiment investigating the effects of oral OXT on visual attention results showed that compared with oral PLC it increased anti-saccade error rates to social and non-social cues, but only increased response latencies to social cues, with similar treatment effects across the different face emotions. Plasma OXT concentrations were again significantly increased 30 min after oral OXT administration and overall percentage concentrations changes were positively associated with anti-saccade errors. Finally, a comparison analysis with previous studies reporting effects of intranasal (Xu et al., 2019) and lingual (Zhuang et al., 2022) administration of OXT revealed no significant differences between the three routes of administration on either anti-saccade error rates or response latencies suggesting that they had similar effects on top-down attention. Overall, the findings from this study support the therapeutic potential for oral administration of OXT using a medicated lollipop approach both for increasing peripheral OXT concentrations and for influencing top-down attention.

Both a validation pilot experiment and the main experiment confirmed that oral administration of OXT using a medicated lollipop approach significantly increased plasma OXT concentrations. The pilot study, where samples were taken at 15 min intervals for 2 h after oral administration, showed that plasma OXT concentrations rose significantly after 15 min and peaked at 30 min (around a 50% increase) but thereafter returned to baseline. In the main experiment plasma OXT concentrations were also increased at 30 min after administration. Parallel measurements in saliva revealed an expected very large increase in OXT concentrations after 30 min (30-fold) which remained increased after 2 h (5-fold), confirming the efficacy of the approach for producing large and long-lasting increases in the oral cavity and thus also influencing blood levels for a long period. Comparisons of the magnitude and time-course of increased OXT concentrations across the same dose (24IU) administered via oral, lingual and intranasal routes revealed no difference between oral and lingual although intranasal administration produced the greatest initial concentration changes (after 15 min). While peripheral basal OXT concentrations may not reliably reflect those in the brain, a recent meta-analysis has shown that peripheral changes evoked by both stress and intranasal OXT administration do more accurately reflect similar changes in the brain (Valstad et al., 2017).

The anti-saccade paradigm used in the current and previous studies can reveal treatment effects on both top-down (shifting attention away from a stimulus–indexed by anti-saccade errors and latencies) and bottom-up attention (shifting attention toward a stimulus–indexed by pro-saccade errors and latencies). Consistent with the previous results for intranasal (Xu et al., 2019) and lingual spray (Zhuang et al., 2021) administration, OXT modulated top-down attention by increasing anti-saccade error rates for both social and non-social stimuli but only increasing anti-saccade latencies for social stimuli. In support of our hypothesis, there was also a positive overall association between changes in plasma OXT concentrations and anti-saccade errors, suggesting that they reflect functional effects in line with our previous study (Kou et al., 2021). However, interestingly the changes in plasma OXT concentrations were more significantly correlated with anti-saccade errors in the PLC than in the OXT group, suggesting that the pattern of endogenous OXT changes in the blood preceding the anti-saccade task are also influencing task performance. Both marked increases and decreases in endogenous levels were observed in some individuals in the PLC group (see Figure 3A), due perhaps to stress-related effects of blood sampling and/or anticipation of performing the eye-tracking task. Indeed, a meta-analysis has reported that stronger associations between OXT and cortisol concentrations occur in individuals anticipating having to perform a task (Brown et al., 2016). Overall, these findings suggest that OXT can interfere with control of top down attention for both social and non-social stimuli in terms of accuracy (Hovey et al., 2020) but its overall influence is strongest for social stimuli (face emotions), evidenced by increased response latencies, indicating an overall greater impact of social stimuli on weakening top-down attention control. Thus, OXT administration makes it more difficult for individuals to shift their attention away from social stimuli in particular. This finding is consistent with those from a previous studies using other paradigms (Averbeck, 2010; Yao et al., 2018).

Oral OXT also significantly reduced post-task SAI scores similar to our previous findings with lingual (Zhuang et al., 2022) and intranasal (Xu et al., 2019) administration and scores were significantly different between the OXT and PLC groups after the task. There were also significant reductions in PANAS mood scores in both OXT and PLC groups, which may reflect experiment fatigue. It is still unclear whether post-task reductions in state anxiety we have observed following OXT treatment in the current study as well as following intranasal and lingual administration (Xu et al., 2019; Zhuang et al., 2022) are due to an anxiolytic effect of the peptide *per se* or some form of interaction with the anti-saccade task. However, in confirmation of findings with lingual administration of OXT (Zhuang et al., 2022) we found no significant correlations between SAI scores and performance on the attention task and thus the two effects of OXT may be independent. Future experiments investigating anxiolytic effects of oral OXT will need to use other more typical paradigms specifically designed for inducing increased anxiety.

A comparative analysis of the effects the three routes of OXT administration on increased anti-saccade error rates and response latencies did not reveal any significant route-dependent differences. A Bayes analysis confirmed the lack of route-dependent significant differences with a moderate fit. This suggests that oral administration of OXT via a medicated lollipop has a similar effect on top-down attention as intranasal and lingual sprays. Similarly, the reduction in state anxiety following oral OXT was also similar across the three different routes of administration and confirmed by Bayes analysis showing a moderate fit. Thus, overall the functional effects of exogenously administered OXT on both top-down attention and state anxiety appear to be route-independent.

One minor difference between the results of the current experiment and those previously reported following intranasal and lingual oxytocin is that more anti-saccade errors were made in response to the non-social stimuli. The non-social stimuli in the current experiment were adapted to better match the stimulus complexity with the face expressions used and this appears to have increased the difficulty in shifting attention away from them. However, most importantly the modulatory effects of OXT were similar to those found after intranasal (Xu et al., 2019) and lingual (Zhuang et al., 2022) administration.

A major topic of discussion regarding the functional effects of exogenously administered OXT concerns the route(s) by which functional effects are produced. On the one hand, intranasal OXT can enter the brain directly through the olfactory and trigeminal nerves to exert its functional effects (see Yao and Kendrick, 2022). However, it can also produce functional effects after entering the peripheral blood system through the highly vascularized nasal cavity (as well as dripping down into the oral cavity). Following entry into the peripheral blood system, OXT may potentially influence brain and behavior either by binding to RAGE and crossing the BBB or via stimulation of vagal projections via its receptors in the heart and gastrointestinal system (Quintana et al., 2021; Yao and Kendrick, 2022), although this remains to be fully established. On the other hand, administration of OXT via oral or lingual spray routes can only produce functional effects via increasing concentrations in peripheral blood (Tabbaa and Hammock, 2020; Yao and Kendrick, 2022). Given that all three delivery routes have in common only peripherally mediated effects, and produce very similar effects on top-down attention and anxiety, this argues strongly that endogenously administered OXT is acting primarily by increasing peripheral concentrations. This is supported by a previous finding that both intranasal (Xu et al., 2019) and lingual (Zhuang et al., 2022) administration of OXT can influence neural responses to emotional faces. However, in this case the patterns of functional effects observed following intranasal and lingual administration on both brain and behavior were different, suggesting that exogenously administered OXT may exert some distinct functional effects following direct entrance into the brain or indirectly after entering peripheral blood. The temporal and spatial patterning of influences on regional OXT receptors via these different routes will also be somewhat different. Thus, it is possible that the route via which exogenously administered OXT produces functional effects may be influenced by both pharmacodynamic factors such as dose (Spengler et al., 2017; Quintana et al., 2021) or task modality (Horta de Macedo et al., 2014) or stimulus salience (Koch et al., 2014; Hovey et al., 2020). Future studies will need to further clarify dose, task and context-dependent influences on effects of direct- compared to indirectly mediated functional effects of exogenously administered OXT on both brain and behavior.

The present study has several limitations. First, only male participants were included in line with previous studies investigating effects of intranasal and lingual oxytocin (Xu et al., 2019; Zhuang et al., 2022), and to avoid potential influences of the menstrual cycle on OXT concentrations. However, sex-dependent differential effects of OXT have been found in several previous studies (Rilling et al., 2014; Gao et al., 2016; Bredewold and Veenema, 2018), so future studies will need to examine effects of oral OXT in female subjects. Secondly, while post-task reductions in state anxiety levels were consistent across different routes of OXT administration future experiments need to confirm anxiolytic effects using other tasks. Thirdly, since positive associations between task performance and changes in plasma OXT following oral treatment occurred in both the OXT and PLC groups, particularly in the latter, it is difficult to differentiate between endogenously and exogenously derived functional effects which would require larger sample sizes and a dose-response approach. Lastly, the sample sizes of the groups in the exploratory comparative analysis of peripheral OXT concentrations were small, and the levels of peripheral OXT concentrations and their trends under the three routes of administration need validation in larger sample sizes.

In conclusion, the current study has validated the use of an oral administration route for OXT using a medicated lollipop approach both for increasing blood and saliva concentrations of OXT and for producing functional effects on top-down attention. Importantly, the effects of oral OXT both on top-down attention and state anxiety were similar to those found following intranasal or lingual administration routes and suggest its potential use therapeutically, particularly in young children in the context of autism. Additionally, these findings support the view that important functional effects of exogenously administered OXT can be mediated via increased peripheral concentrations as opposed to via direct entry into the brain which can only occur via an intranasal route.

## Data availability statement

The raw data supporting the conclusions of this article will be made available by the authors, without undue reservation.

## Ethics statement

The studies involving human participants were reviewed and approved by the University of Electronic Science and Technology of China Ethics Committee. The patients/participants provided their written informed consent to participate in this study.

## Author contributions

KK designed the study and provided the critical revisions. DX and QL collected the data. DX and YZ completed the ELISA analysis. DX, QZ, SY, and WZ analyzed the data. DX drafted the manuscript. All authors contributed to the article and approved the submitted version.

## Footnotes

1
https://jasp-stats.org
