# Supplementary material for: Oro-mucosal administration of oxytocin using medicated lollipops alters social attention, similar to intranasal and lingual routes: Implications for therapeutic use
Source: Front Neurosci. 2022 Oct 25;16:1022101. doi: 10.3389/fnins.2022.1022101 (PMC9641372; doi:10.3389/fnins.2022.1022101)
Supplement: Supplementary file 1 [file Data_Sheet_1.docx]

Supplementary Material


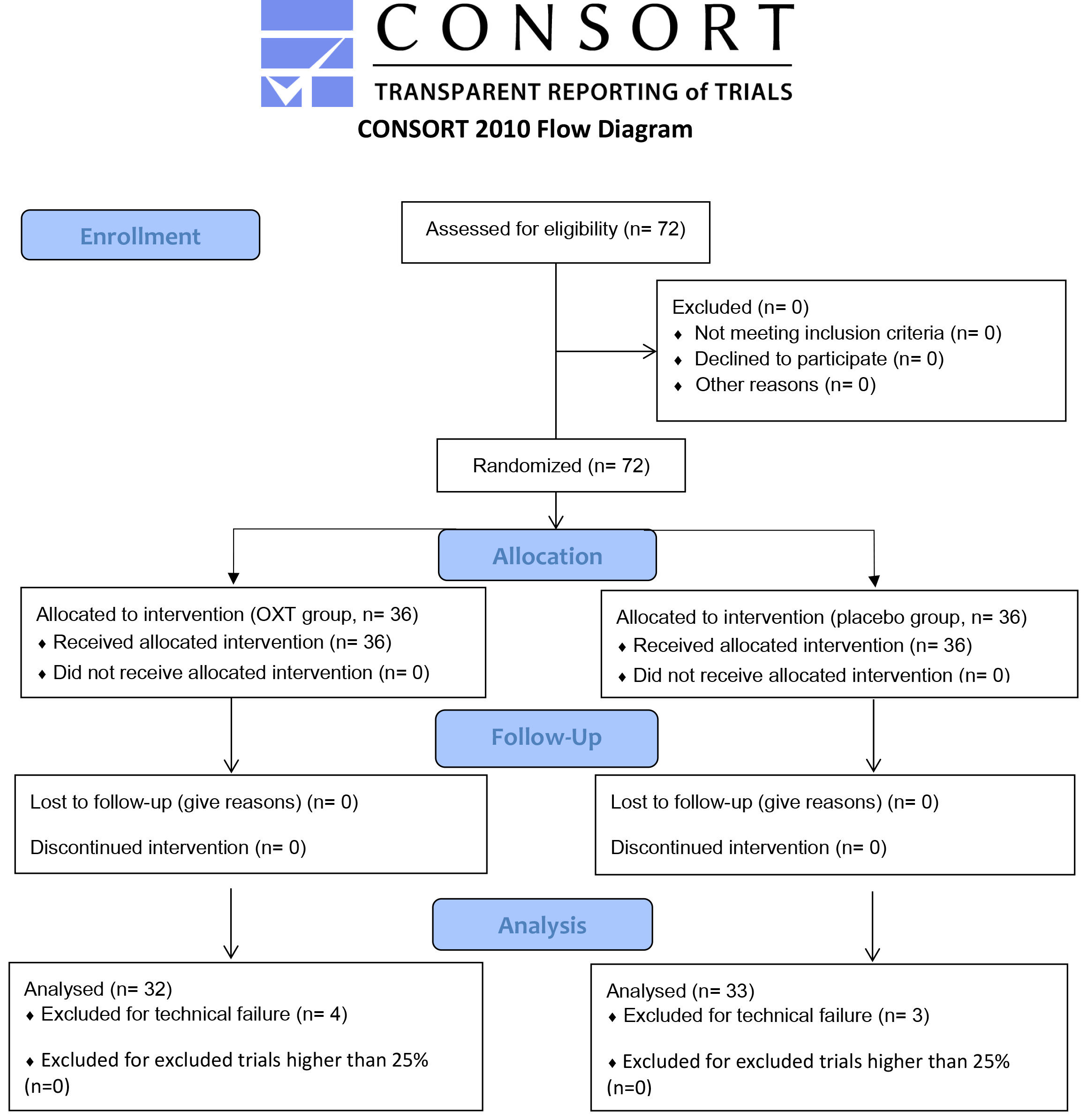


**Figure. S1** Consolidated standards of reporting trials (CONSORT) flow chart.


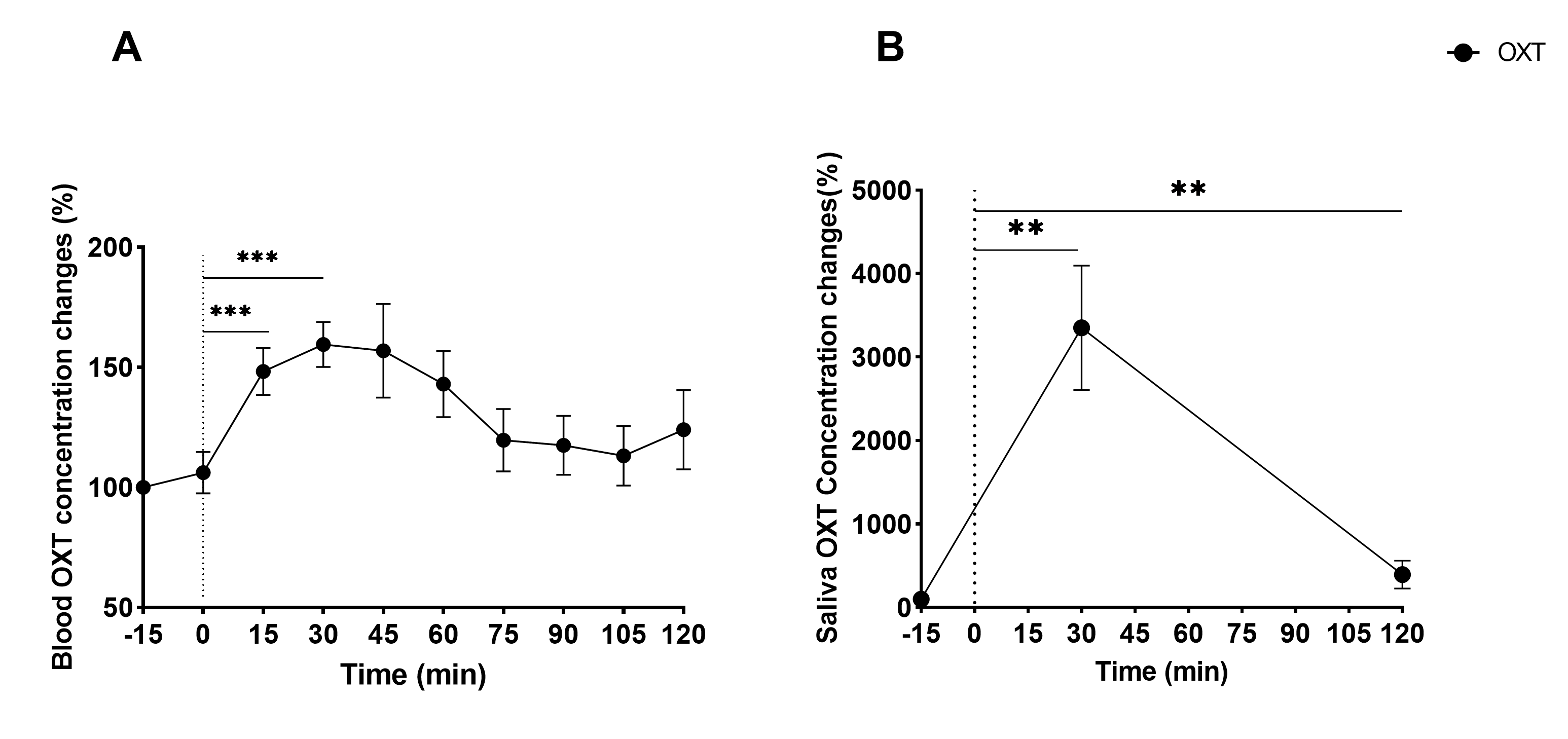


**Figure. S2** the plasma and saliva oxytocin concentration levels before and for 120 minutes after treatment. **(A**) The concentration of oxytocin in the blood peaks 30 minutes after administration and then gradually decreases. (**B**) Salivary oxytocin concentrations followed the same trend as those in blood but remained significantly higher than baseline two hours after treatment. Mean ± SD baseline OXT concentrations in plasma were 8.54 ± 2.92 pg/ml and 8.80 ± 4.58 pg/ml in saliva.

*: *p_Bonferroni_* < 0.05, **: *p_Bonferroni_* < 0.01, ***: *p_Bonferroni_* < 0.001
